# The Impact of Chemotherapy on Arterial Stiffness and Ventricular–Arterial Coupling in Women with Breast Cancer

**DOI:** 10.3390/ph17091115

**Published:** 2024-08-23

**Authors:** Nikolaos P. E. Kadoglou, Alexandriani Dimopoulou, Irene Tsappa, Pampina Pilavaki, Anastasia Constantinidou

**Affiliations:** 1 Medical School, University of Cyprus, Nicosia 2029, Cyprus; 2 Department of Medical Oncology, Bank of Cyprus Oncology Centre, Nicosia 2029, Cyprus

**Keywords:** chemotherapy, arterial stiffness, cardio–ankle vascular index, global longitudinal strain, breast cancer, ventricular–arterial coupling

## Abstract

Background: The cardiac toxicity of chemotherapy for breast cancer is not uncommon and has been associated with elevated morbidity and mortality. In the present study, we assessed the impact of chemotherapy on cardiovascular function by assessing the cardio–ankle vascular index (CAVI), global longitudinal strain (GLS) and ventricular–arterial coupling (VAC: CAVI/GLS ratio) in chemotherapy-treated women. Methods: This prospective study enrolled 78 women with breast cancer who were receiving anthracycline-based chemotherapy +/− anti-HER2 therapy (trastuzumab +/− pertuzumab). Forty-one age-matched healthy women served as controls. We comparatively evaluated left ventricular ejection fraction (LVEF), CAVI, GLS and VAC, between the chemotherapy and control groups. We also assessed their changes over time (baseline, 3-month and 6-month time point) and their independent association with the incidence of cancer therapy-related cardiovascular dysfunction (CTRCD) in the chemotherapy group. Results: In comparison to healthy controls, women receiving chemotherapy presented with significantly higher GLS (from −21.02 ± 2.09% to −19.01 ± 2.81%, *p* < 0.001) and VAC (−0.36 ± 0.06 to −0.41 ± 0.11, *p* < 0.001). The presence of CTRCD was associated with a further increase in GLS and CAVI and a significant decline in LVEF and VAC compared to CTRCD-free women (*p* < 0.001). Baseline, CAVI, GLS and VAC were independently associated with CTRCD development during follow-up. Conclusion: Women with breast cancer undergoing chemotherapy displayed abnormal levels of CAVI, VAC and GLS, compared to healthy individuals. Those effects on VAC and CAVI were more exaggerated among women with CTRCD, implicating their potential use to refine screening and therapeutic strategies for this specific population.

## 1. Introduction

Anthracycline-based chemotherapy is still the cornerstone of breast cancer patients, unless clinically contraindicated, leading to unambiguous improvement in survival rates [1]. Roughly 20% of breast cancers are human epidermal growth factor receptor 2 (HER2)-positive [2]. In those patients, trastuzumab, a monoclonal antibody, has been incorporated into breast-cancer treatment, and it is used sequentially following anthracyclines. Even in trials examining the efficacy of trastuzumab, almost 80% of participants received anthracycline-based chemotherapy [3]. The adverse impact of chemotherapy on myocardial function has been extensively described in the recent European Society of Cardiology Guidelines and related analysis [4]. Long-term results in breast-cancer survivors have reported the development of cardiovascular diseases in up to 16% of chemotherapy receivers in a 10-year follow-up period [5]. The early detection of the adverse impact of anthracyclines and/or trastuzumab administration in patients with breast cancer on myocardial dysfunction is of paramount prognostic value [6,7]. Speckle tracking in echocardiography and its index, global longitudinal strain (GLS), has been proposed as a more sensitive index of myocardial dysfunction at early stages in breast-cancer patients receiving chemotherapy [8,9]. Therefore, GLS monitoring has been established as an effective approach in those patients.

In parallel, there is growing evidence of a negative impact of chemotherapy on arterial stiffness in patients with malignancies [10]. In the case of breast cancer, anthracyclines and trastuzumab seem to increase arterial stiffness as well [11,12]. Regarding its independent association with future cardiovascular diseases [13], arterial stiffness could serve as a prognostic index of cardiovascular system impairment in breast-cancer patients under chemotherapy. However, the underlying mechanisms of arterial stiffening after chemotherapy remain mostly elusive. Pulse-wave velocity (PWV) constitutes a non-invasive, simple measurement of arterial stiffness, and its measurement is easy and repeatable. Notably, its main disadvantage is the high dependence on blood pressure [14]. To overcome this drawback, another formula of PWV calculation, termed the cardio–ankle vascular index (CAVI), has been proposed [15]. We and other investigators have documented the power of CAVI in patients with [16,17] and without cardiovascular diseases [18]. Despite previous evidence in other populations, there are no data about the relationship of subclinical vascular damage assessed by CAVI with the chemotherapy in women with breast cancer.

The role of ventricular–arterial coupling (VAC) in the pathophysiology of CVDs has long been described [19]. Increasing evidence supports its prognostic power in CVD [20]. Very limited data have indicated VAC impairment due to anti-cancer chemotherapy [21]. Regarding the sensitivity of VAC to promptly detect cardiovascular dysfunction, it is noteworthy to examine the clinical application of it in cancer patients under chemotherapy. Current data are scarce [22], and several formulas have been proposed for VAC calculation. Most of them are not easily applicable in daily clinical practice, and cut-off values are absent. Therefore, the interpretation of VAC results is challenging. Most recently, the PWV/GLS ratio has emerged as a promising, easily repeatable measure of VAC [22]. It has been tested as a prognostic marker of left ventricular dysfunction [23] or organ damage [24] in patients with myocardial infarction and hypertension, respectively. From the theoretical point of view, the arterial stiffening and the concomitant cardiac dysfunction induced by chemotherapy will lead to VAC impairment. Thereby, VAC could become ideally a sensitive and objective marker of cardiovascular dysfunction developed in chemotherapy-treated patients. However, there is little evidence supporting the negative impact of chemotherapy on VAC in cancer patients [25]. More and larger studies are required to validate whether chemotherapy impairs VAC in cancer patients [26]. The validation process has two significant limitations: previous studies included a great variance in cancer types, and methods of VAC calculation showed high diversity, since there is still no single widely accepted methos [27]. Our study focused on breast-cancer patients and used a modern formula of VAC calculation with less drawbacks compared to previous studies.

The aim of this study was to assess the alterations in arterial stiffness by calculating CAVI, after therapy with anthracyclines, either with or without anti-HER2 therapy (trastuzumab +/− pertuzumab) in women with breast cancer. In addition, we assessed GLS at echocardiogram and calculated VAC changes during the follow-up.

## 2. Results

### 2.1. General Characteristics

We initially enrolled 84 women with newly diagnosed or metastatic breast cancer treated with anthracycline based-chemotherapy with or without anti-HER2 therapy (trastuzumab +/− pertuzumab). In particular, 70 patients had early breast cancer: 18 were HER2+ and received trastuzumab treatment, 6 were triple-negative and all received anthracycline-based chemotherapy. The remaining were ER+ HER2−. There were 8 metastatic patients, of which one was HER2+, one was triple negative and three were ER+ HER2−.

During 6-month follow-up, three patients died due to cancer, two were lost from follow-up and one denied the second visit for personal reasons. At the end, 78 women completed all measurements and were analyzed, while 41 healthy women served as controls (2:1 ratio). At baseline, there were no significant differences between groups across all demographic and hemodynamic parameters (Table 1). A small percentage of women in the chemotherapy group had hypertension (8.5%) and/or dyslipidemia (6.1%) and only one woman was treated for diabetes. Notably, among hypertensive women, only half of them (four patients) were already on anti-hypertensive medications. In parallel, half of participants in both groups at baseline were already in the menopause period. We did not expect any impact of menopause on their cardiovascular system, since the vast majority of women had recently entered the menopause period. No significant differences were seen regarding the CAVI, GLS and VAC between groups at baseline (*p* > 0.05; Table 1).

### 2.2. Follow-Up Results

Significant reductions in BMI (from 27.51 ± 6.68 kg/m^2^ to 26.02 ± 6.42 kg/m^2^) and VAC (−0.36 ± 0.06 to −0.41 ± 0.11, *p* < 0.001) were documented between baseline and the 6-month time point, while GLS considerably increased (from −21.02 ± 2.09% to −19.01 ± 2.81%, *p* < 0.001) in the chemotherapy group. Notably, there was a marginal non-significant reduction in LVEF (from 63 ± 9% to 59 ± 8%, *p* = 0.098) and a trend toward increased CAVI (from 7.10 ± 1.25 to 7.61 ± 1.45, *p* = 0.157).

During the follow-up, 20 patients achieved the CTRCD criteria. No acute cardiovascular events or cardiac-related hospitalization were reported during the follow-up period. At baseline, patients developing CRTCD did not differ from the CRTCD-free patients. However, at the end of the study, the CTRCD subgroup appeared to have a significant decline in LVEF and VAC and a remarkable increase in GLS and CAVI in comparison to the CTRCD-free patients and healthy controls (*p* < 0.001). No significant differences were detected between those groups in the rest of examined parameters, like changes in blood pressure or BMI. Notably, CTRCD-free patients showed a slight deterioration in CAVI, GLS and VAC, but those changes did not achieve a significant level.

The vast majority of smokers complied with oral instructions for smoking cessation, so the smoking rate dramatically decreased in both groups at follow-up. At the end of follow-up, one hypertensive woman stopped anti-hypertensive medications due to low levels of blood pressure, and another one developed hypertension and ultimately required therapy. Overall, the number of hypertensive women remained unaltered, and the same was true for their pharmaceutical regimen. Table 2 summarizes the measurements of CRTCD vs. CRTCD-free patients at baseline and the end of the study.

### 2.3. Correlations

We examined all possible correlations with CTRCD development with all variables (anthropometric, clinical, echocardiographic and pharmaceutical) already listed in the tables at the end of the study. The values of CAVI, GLS and VAC significantly correlated with CTRCD development. LVEF was not included since it was almost the single major criteria for CRTCD identification. Medications were examined as covariates. Those parameters entered the linear regression analysis, and all of them were independently associated with CRTCD occurrence (Table 3).

## 3. Discussion

The main findings of this prospective, observational study in female patients with breast cancer but without CV risk factors undergoing chemotherapy were (1) the significant decline in GLS; (2) the considerable elevation in CAVI, an index of aortic stiffness; and (3) the significant reduction in VAC, as expressed by the CAVI/GLS ratio after 6 months. Those effects were observed in the whole cohort, but they were predominantly accompanied by CTRCD development.

In agreement with the results of previous studies, a 6-month chemotherapy treatment with anthracyclines and/or anti-HER2 therapy considerably reduced GLS in the whole cohort of women with breast cancer who were undergoing chemotherapy, while an almost 20% of the cohort experienced CTRCD. The latter subgroup showed the most resounding decline in GLS, associated with significant impairment in LVEF. However, the rest of women—who were without clinically or echocardiographically evidenced CTRCD—showed a small but statistically significant reduction in GLS as well, compared to the baseline and controls, implicating a subtle systolic dysfunction. GLS is capable of unmasking even subtle reductions in cardiac output [28]. The accumulated data support the high incidence of cardiac dysfunction induced by chemotherapy, even when it is not clinically apparent or the LVEF reduction is slight [29,30]. The clinical significance of the GLS decline is paramount since it has been linked with an adverse prognosis [31]. Therefore, early detection and prompt therapy may prevent the deterioration of cardiac systolic function and thereby improve the prognosis of cancer patients [6].

Up to now, there have been scarce data on the impact of anti-cancer chemotherapeutic agents on the vascular system, showing a potential “vascular toxicity”. Previous studies have demonstrated increased PWV after chemotherapy in breast-cancer patients receiving anthracyclines [11], but not trastuzumab [12], in the long-term. Therefore, anthracyclines may impair endothelial function and precipitate atherosclerosis in large arteries, predisposing patients to adverse cardiovascular events. This is the first study reporting the impact of chemotherapy on vascular function using CAVI, avoiding the interference of blood pressure.Vascular toxicity was previously implicated in a similar group of patients [32]. Contrary to studies on hypertensive patients, our study provides more robust evidence of the adverse effects of chemotherapy on the elasticity of arteries, since the blood pressure-lowering pharmaceutical agents remained unaltered in chemotherapy recipients. Therefore, the early diagnosis of chemotherapy-induced vascular toxicity should be the first priority, especially when classic cardiovascular risk factors remain “well-controlled” in women who have breast cancer and are under chemotherapy. The development of vascular cardiac injury, either with or without CTRCD occurrence, may not only affect chemotherapy continuation but also the short-term prognosis. The incorporation of CAVI measurement as a routine clinical practice of monitoring patients under chemotherapy could add a sensitive, non-invasive and objective tool to promptly identify patients at an early stage of CTRCD and tailor their therapy. Future studies will clarify the prognostic value of our findings.

The mechanical coupling of LV with the aorta demonstrates how the aortic-stiffness affects the LV function leading to both systolic and diastolic dysfunction, and eventually to the adverse remodeling of LV which follows the development of heart failure [33,34]. Regarding VAC as the crosstalk between the left ventricular function and arterial system, a plethora of studies have proposed it as an index of cardiovascular performance in several conditions without established CVD but at high risk for cardiovascular morbidity and mortality, like hypertension, diabetes mellitus, etc. [35,36]. Notably, the prognostic value of VAC has also been confirmed in patients with established CVD, like those after recent myocardial infarction [37]. There is a growing body of evidence supporting the usage of VAC as a therapeutic target that can help ameliorate the prognosis. In the context of acute heart failure, diuretics administration may improve arterial elasticity, rather than cardiac contractility, and it may yield significant benefits in the post-discharge period [19]. The pharmaceutical modification of VAC and its link with cardiovascular outcomes remain under investigation. Despite the pathophysiological link of VAC with cardiovascular alterations in several conditions, its major limitation remains the establishment of a gold-standard technique for its measurement [38]. The ideal method should be easy to perform and feasible, with high reproducibility and the ability to be validated.

Several indices have been proposed for VAC assessment. Some investigators have used the PWV/GLS ratio for VAC calculation, trying to limit potential confounders as much as possible. A reduced ratio has been linked with clinical outcomes in a small spectrum of cardiovascular disease [39], but, most importantly, it has been associated with greater severity of HF and a worse functional capacity [40]. In our study, we used CAVI as an index of arterial stiffness, as it demonstrates greater advantages than PWV. It remains to be proved whether this novel ratio represents the VAC. From the pathophysiology perspective, an altered CAVI/GLS ratio may reflect the net result of a cluster of disorders, including endothelial dysfunction, renin–angiotensin-system activation, abnormal collagen turnover, increased metalloproteinase activity and fibrosis, contributing to both cardiac and vascular dysfunction. Our study clearly demonstrated aortic stiffening and reduced VAC in patients with CTRCD, indicating an interplay between cardiac and vascular function. After extrapolating data from other clinical conditions, we noted that an impaired VAC adversely affects the LV afterload, with apparent consequences like higher LV work and oxygen-supply demands [41]. We hypothesized that the cardiovascular toxicity of chemotherapeutic agents may not only explain their lower exercise capacity, but it can potentially predict their higher cardiovascular risk. Hence, CTRCD cannot entirely describe the cardiovascular complications of chemotherapy. Several cardiac alterations have been described in the past after anthracyclines or adjuvant therapy with trastuzumab, leading to a worse prognosis [42,43]. Therefore, the VAC measurement may unmask another phenotype of cardiovascular toxicity before the cardiac dysfunction becomes apparent. This may also explain the high cardiovascular risk of chemotherapy recipients in the long term in relation to myocardial injury and/or coronary artery disease.

There are several drawbacks to the present study. The most important of them is the small sample size. This was a small single-center study, which included a homogeneous cohort of female patients with breast cancer, and our preliminary findings cannot be generalized. The limited number of participants may also explain the existence of a few correlations between variables when the studied parameters were statistically different between the two groups. Therefore, a larger sample size is necessary to obtain robust evidence and detect further associations. Moreover, the cross-sectional design of our study prevented us from drawing firm conclusions about causality. Regarding the fact that some cardiotoxic effects may first become apparent after 6 months of follow-up, longer protocols should be carried out to better define and investigate the predictive value of arterial stiffness and VAC in women with breast cancer, as well as the impact of treatment on them.

In conclusion chemotherapy deteriorated arterial elasticity and reduced VAC in women with breast cancer during a 6-month follow-up. Therefore, this group has the potential for accelerated atherosclerosis and higher cardiovascular risk.

## 4. Materials and Methods

### 4.1. Research Design

This is a prospective case–control study comparing women with breast cancer who are eligible for chemotherapy (chemotherapy group) and healthy women (control group). At baseline, all participants underwent a medical history recording, clinical examination, arterial stiffness assessment and echocardiography to obtain measurements. All of those exams are described in detail in the following subsections and were performed before chemotherapy initiation. After the initial assessment, only women with breast cancer were followed up at 3 and 6 months. At those time-points, arterial stiffness and echocardiography measurements were obtained. Moreover, the chemotherapy group was further subdivided into subgroups of patients developing cancer therapy-related cardiovascular dysfunction (CTRCD) during the follow-up and CTRCD-free subgroup.

**Chemotherapy group:** We consecutively recruited 84 women with breast cancer who were eligible for chemotherapy. All women with breast cancer received anthracycline based-chemotherapy (FEC, AC or EC with or without Paclitaxel/Taxotere) either plus or not with anti-HER2 therapy (trastuzumab +/− pertuzumab).

**Control group:** A group of 41 age-matched healthy women without cancer, cardiovascular diseases or other chronic diseases which may affect the studied parameters served as the control group at baseline. The latter group was enrolled after an open invitation was sent to family members or friends of patients with breast cancer to participate in our study. Such chronic diseases included hypertension, diabetes, kidney (creatinine ≥2 mg/dl) or liver failure (AST ≥ 3 times the upper normal limit), systemic inflammatory diseases or autoimmune diseases, atrial fibrillation, acute infection or surgery.

**Subgroups based on CTRCD development:** According to current guidelines, we considered cancer therapy-related cardiovascular dysfunction (CTRCD) in asymptomatic patients with (1) new LVEF reduction <40%; (2) new LVEF reduction to 40–49%, when the amount of LVEF exceeds ≥10%; (3) new LVEF reduction to 40–49%, when the amount of LVEF is less than 10% and concomitant new relative decline in GLS by 15% from baseline; and (4) LVEF ≥ 50% and new relative decline in GLS by >15% from baseline. Biomarkers were not available to most patients, and they were not included in the analysis. We considered HF symptoms as an additional index, only when echocardiography findings were compatible to CRTCD, because fatigue and dyspnea were common complaints of patients under chemotherapy and could not distinguish those with CTRCD. When the latter diagnosis was set, the patients were referred to the cardio-oncology outpatient clinic for monitoring and HF-related therapy commitment. So, they were not further included in our study.

Written informed consent was obtained from all participants before enrollment. All procedures were performed according to the principles of Helsinki Declaration and were approved by the National Bioethics Committee of Cyprus (reference number of approval: EEBK/EEP/2021/34).

### 4.2. Participants’ Clinical Examination

At each visit, we measured participants’ weight to calculate the BMI and the blood pressure after sitting for 5 min. Based on a structured questionnaire, we recorded medications and co-morbidities, defined as follows: hypertension, BP ≥ 140/90 mmHg measured on repeated occasions or receipt of antihypertensive drugs; hyperlipidemia, fasting serum low-density-lipoprotein cholesterol (LDL-C) ≥130 mg/dL or statin therapy; active smokers, current or within the previous 6 months; diabetes mellitus, fasting plasma glucose (FPG) ≥ 126 mg/dL, or HbA1c ≥ 6%, or antidiabetic drugs; and coronary artery disease, history of stable or unstable angina, myocardial infarction, percutaneous or surgical myocardial revascularization.

### 4.3. Arterial-Stiffness Assessment

The arterial stiffness of the participants was calculated as the cardio–ankle vascular index (CAVI) (Vasera VS-1500, Fukuda Denshi, Tokyo, Japan). The calculation of the CAVI was based on the following formula: CAVI = a × PWV2/SPB−DPB + b (a and b are constants; PWV, pulse-wave velocity from the heart to the ankle; SBP, systolic blood pressure; and DBP, diastolic blood pressure). CAVI is a non-invasive and reproducible technique independent of blood pressure proportionally related to a higher risk of cardiovascular diseases. According to the recommended procedure, the participants were in a supine position for relaxation for 10 min. Four electrodes were attached to the participants’ limbs for electrocardiogram (ECG) recording, and a microphone placed on participants’ chest recorded phonocardiogram (PCG). Blood pressure measurements were obtained from cuffs wrapped around the four limbs. Basically, CAVI was calculated by recording the distance from the level of the aortic valve (i.e., brachial level) to each ankle and the time delay from the aortic valve closing to the detected notch in arterial pressure wave at unilateral ankle [44]. The right and left CAVI were measured and then averaged. Besides this, the device provided the ankle–brachial index (ABI) for both sides.

### 4.4. Global Longitudinal Stain (GLS) and Ventricular–Arterial Coupling (VAC)

Using a standard imaging system (GE E95 ultrasound scanner, Horten, Norway), two independent experienced cardiologists performed the echocardiographic studies at baseline and during follow-up. The offline imaging analysis was performed on a single PC workstation by both those cardiologists blinded to patients’ data (GE Echopac 205 version software). Standard echocardiographic measurements included LVEF, LV end-diastolic volume (LVEDV) and LVESV. The LV myocardial deformation was measured using the LV global longitudinal stain (GLS) formula. In brief, analyzing the 3 apical views of echocardiography, the longitudinal strain was measured, each wall was subsequently divided into 3 segments (basal, mid and apical), and a total of 17 segmental strain curves were obtained. GLS was calculated as the average value of all strain peak values. The inter-observer variability was based on the intra-class correlation coefficient (ICC), which showed a high reproducibility [0.94 (0.84–0.97)]. The intra- and inter-observer reliability of strain analysis by our group was previously reported, and it is very low (<2.5%). Besides this, we calculated the echocardiography-derived myocardial work at rest by entering the blood pressure measurements to GLS analysis. Using vendor’ software (GE echopac 205 version), we calculated the parameters of myocardial work. We then calculated the CAVI/GLS ratio as an index of VAC.

### 4.5. Statistical Analysis

Normally distributed continuous variables were presented as the mean ± SD. Normality of distribution was assessed by the Kolmogorov–Smirnov test. Comparisons of continuous and categorical variables were analyzed with Student’s *t*-test and chi-square test, respectively. Changes in continuous variables within and between groups were assessed using paired samples and Student’s *t*-tests, respectively. To test the univariate and multivariate associations of CAVI with any of the study population characteristics, we performed a Pearson correlation and multiple linear regression analysis for normally distributed variables. Medications were examined as covariates. A two-tailed *p* < 0.05 was considered to be significant. The computer software package SPSS (version 25.0; SPSS Inc., Chicago, IL, USA) was used for statistical analysis.

## 5. Conclusions

In conclusion, the combined therapy of anthracyclines plus trastuzumab in women with breast cancer reduced both GLS and VAC values and increased the arterial stiffness assessed by CAVI. Those effects were more pronounced among women developing CRTCD within 6 months from chemotherapy initiation. The clinical impact of cardiac and vascular toxicity of these chemotherapeutic agents and their potential consequences remains to be proved in the long term.

## Figures and Tables

**Table 1:** Comparison between groups at baseline.

Variables	Chemotherapy Group (N = 78)	Control Group (N = 41)	*p*-Value
Age (years)	53 ± 11	51 ± 6	0.121
Hypertension (n)	7 (8.9%)	0	-
Dyslipidemia (n)	5 (6.4%)	0	-
Diabetes (n)	1 (1.3%)	0	-
Smokers (n)	13 (16.7%)	6 (15%)	0.777
Menopause (n)	41 (52.6%)	19 (46.3%)	0.809
CAVI	7.10 ± 1.25	6.99 ± 1.05	0.758
GLS (%)	−21.02 ± 2.09	−21.16 ± 1.55	0.633
VAC1	−0.36 ± 0.06	−0.33 ± 0.05	0.290
BMI (kg/m^2^)	27.51 ± 6.68	26.52 ± 4.65	0.156
SBP (mmHg)	131 ± 26	130 ± 13	0.767
DBP (mmHg)	83 ± 10	81 ± 8	0.204
LVEF (%)	63 ± 9	66 ± 7	0.105
E/A ratio	0.98 ± 0.22	1.00 ± 0.38	0.939
E/E’ ratio	7.17 ± 2.75	6.90 ± 2.85	0.806
LAVI (ml/m^2^)	29.55 ± 7.68	29.35 ± 5.50	0.944
TAPSE (cm)	2.32 ± 0.32	2.59 ± 0.42	0.062
RVS’ (m/s)	0.14 ± 0.04	0.15 ± 0.02	0.361
TRVmax (m/s)	2.23 ± 0.38	2.85 ± 3.16	0.232
Pharmaceutical regimen			
ACEI (n)	3 (3.9%)	0	
CCB (n)	1 (1.3%)	0	
Statins (n)	2 (2.6%)	0	

ACEI, angiotensin-converting enzyme inhibitors; BMI, body-mass index; CAVI, cardio–ankle vascular index; CCBs, calcium channel blockers; DBP, diastolic blood pressure; E/A ratio, E transmitral flow velocity/A transmitral flow velocity; E/E’, E transmitral flow velocity/E’ tissue; GLS, global longitudinal strain; LAVI, left atrial volume index; LVEF, left ventricular ejection fraction; n, number; RVS’, right ventricular systolic excursion velocity by tissue Doppler; SBP, systolic blood pressure; TAPSE, tricuspid annular plane systolic excursion; TRVmax, maximal tricuspid regurgitation velocity; VAC, ventricular–arterial coupling. The terms of co-morbidities (hypertension, dyslipidemia and diabetes) are defined in Section 4.2.

**Table 2:** Changes in variables during follow-up within subgroups of CTRCD vs. CTRCD-free patients.

Variables	CTRCD Subgroup (N = 20)		CTRCD-Free Subgroup (N = 58)		*p*-Value
	Baseline	End	Baseline	End	
Age (years)	53 ± 11		51 ± 6		-
Hypertension (n)	2 (10%)	1 (5%)	5 (8.6%)	6 (10.3%)	-
Dyslipidemia (n)	1 (5%)	1 (5%)	4 (6.9%)	5 (8.6%)	-
Diabetes (n)	0	1 (5%)	0	1 (1.8%)	-
Smokers (n)	3 (15%)	1 (5%)	10 (17.2%)	2 (3.2%)	-
Menopause (n)	10 (50%)	-	31 (53.4%)	-	-
CAVI	7.22 ± 1.34	9.51 ± 1.24 *	7.02 ± 0.91	7.51 ± 1.05	<0.001
GLS (%)	−21.01 ± 2.06	−16.43 ± 1.67 *	−21.09 ± 2.08	−20.22 ± 1.73	<0.001
VAC	−0.44 ± 0.09	−0.58 ± 0.09 *	−0.35 ± 0.06	−0.37 ± 0.06	<0.001
BMI (kg/m^2^)	27.79 ± 5.56	26.18 ± 6.89 *	27.34 ± 7.05	26.02 ± 6.42 *	0.887
SBP (mmHg)	137 ± 15	137 ± 15	129 ± 27	129 ± 27	0.995
DBP (mmHg)	86 ± 9	86 ± 9	82 ± 10	82 ± 10	0.996
LVEF (%)	61 ± 4	52 ± 11 *	63 ± 7	61 ± 7	0.076
E/A ratio	0.98 ± 0.22	0.71 ± 0.31	0.99 ± 0.21	0.95 ± 0.20	0.101
E/E’ ratio	7.33 ± 3.16	8.52 ± 3.59	7.09 ± 3.01	7.99 ± 4.16	0.101
LAVI (mL/m^2^)	30.29 ± 9.57	33.13 ± 11.82 *	27.15 ± 7.59	29.76 ± 8.10	0.554
TAPSE (cm)	2.21 ± 0.32	2.41 ± 0.24	2.40 ± 0.25	2.31 ± 0.39	0.675
RVS’ (m/s)	0.13 ± 0.03	0.16 ± 0.37	0.14 ± 0.05	0.14 ± 0.02	0.981
TRVmax (m/s)	2.47 ± 0.52	2.71 ± 0.46	2.10 ± 0.51	2.28 ± 0.30	0.429
Pharmaceutical regimen					
ACEI (n)	1	1	2	2	-
CCB (n)	0	0	1	1	-
Statins (n)	1	1	1	1	-

ACEI, angiotensin-converting enzyme inhibitors; BMI, body-mass index; CAVI, cardio–ankle vascular index; CCBs, calcium channel blockers; DBP, diastolic blood pressure; E/A ratio, E transmitral flow velocity/A transmitral flow velocity; E/E’, E transmitral flow velocity/E’ tissue; GLS, global longitudinal strain; LAVI, left atrial volume index; LVEF, left ventricular ejection fraction; n, number; RVS’, right ventricular systolic excursion velocity by tissue Doppler; SBP, systolic blood pressure; TAPSE, tricuspid annular plane systolic excursion; TRVmax, maximal tricuspid regurgitation velocity; VAC, ventricular–arterial coupling. The terms of co-morbidities (hypertension, dyslipidemia and diabetes) are defined in Section 4.2. * Indicates a p < 0.05 change in variables within groups.

**Table 3:** Associations between CRTCD and other variables within the chemotherapy group.

Variables	Univariate Analysis		Multivariate Analysis	
	β (SE)	*p*	β (SE)	*p*
CAVI	0.663 (0.099)	<0.001	0.268 (0.099)	0.008
GLS	−0.401 (0.051)	<0.001	0.273 (0.043)	0.041
VAC	−0.826 (0.077)	<0.001	−0.701 (0.145)	<0.001

CAVI, cardio–ankle vascular index; GLS, global longitudinal strain; VAC, ventricular–arterial coupling. Most important, VAC, at the end of the study, correlated with CTRCD (r = −0.793, *p* < 0.001) and LVEF (r = 0.496, *p* < 0.001), and those parameters remained independent determinants of VAC.

## Data Availability

The data is unavailable due to ethical restrictions.
